# Supplementary material for: Graphene Infused Ecological Polymer Composites for Electromagnetic Interference Shielding and Heat Management Applications
Source: Materials (Basel). 2021 May 26;14(11):2856. doi: 10.3390/ma14112856 (PMC8198334; doi:10.3390/ma14112856)
Supplement: Supplementary file 1 [file materials-14-02856-s001.zip › materials-1186425-supplementary.pdf]

materials

MDPI

Supplementary Materials

Graphene Infused Ecological Polymer Composites for Electromagnetic Interference Shielding and Heat Management Applications

Klaudia Zeranska-Chudek [1,*], Anna Wróblewska [1], Sebastian Kowalczyk [2], Andrzej Plichta [2] and Mariusz Zdrojek [1]

[1] Faculty of Physics, Warsaw University of Technology, Koszykowa 75, 00-662 Warsaw, Poland; anna.wroblewska@pw.edu.pl (A.W.); mariusz.zdrojek@pw.edu.pl (M.Z.)
[2] Faculty of Chemistry, Warsaw University of Technology, Noakowskiego 3, 00-664 Warsaw, Poland; skowalczyk@ch.pw.edu.pl (S.K.); andrzej.plichta@pw.edu.pl (A.P.)
* Correspondence: klaudia.zeranska@pw.edu.pl

SEM analysis of GNP in form of powder

Figure S1**Error! Reference source not found.** shows SEM pictures of three types of GNP used in this study, with different lateral sizes (0.2, 5 and 25 μm). The pictures are shown at a common magnification to show the difference of flake size.

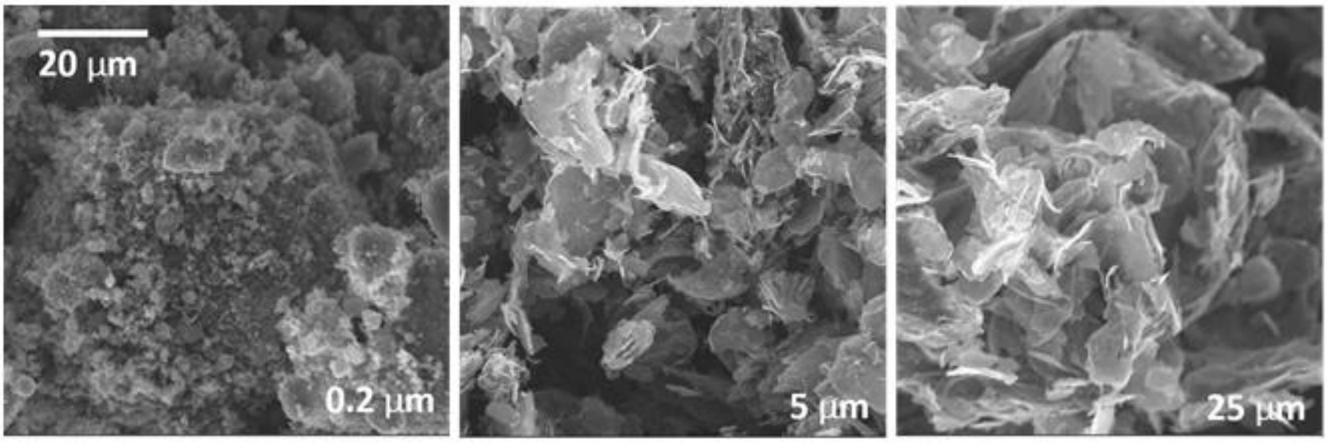

Figure S1. SEM pictures showing GNP of 0.2, 5 and 25 μm lateral size.

Raman spectroscopy analysis of PLA/GNP composites

Figure S2 shows Raman spectra of the PLA/GNP composites. The point of these graphs is to show differences in Raman spectra for composites with the same GNP loading and different GNP lateral sizes (Figure S2(a)) and composites infused with 25 μm GNP with various GNP loadings (Figure S2(b)). Figure S2(b) shows the vanishing of PLA characteristic peaks with the growing GNP concentration.

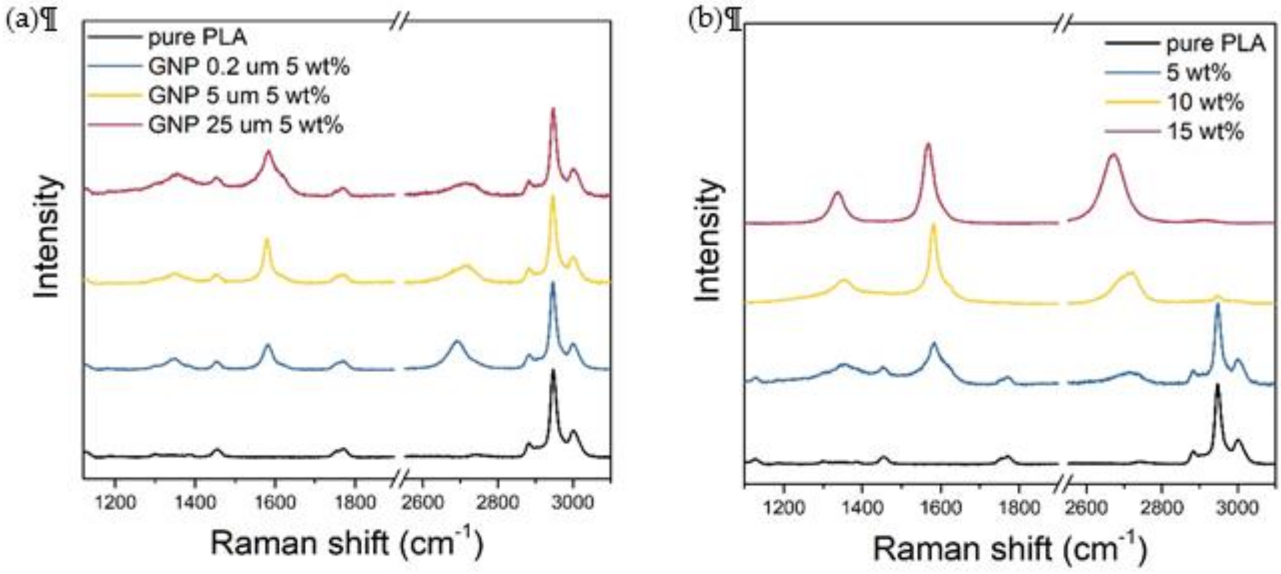

Figure S2. Raman spectra of (**a**) PLA/GNP composites with 0.2, 5 and 25 µm GNP lateral size at 5 wt% loading, and (**b**) PLA/GNP composites of three GNP loading, together with a pure PLA reference.

EMI SE additional information

Figure S3 shows the comparison of total shielding efficiency and its reflectance component of an empty coax line and a pure PLA sample. It is clear the SE_R peak near 12 GHz is characteristic to PLA, as it is not visible in empty coax line spectrum. The coax line loaded with pure polymer shows slightly lower EMI shielding efficiency in the studied range.

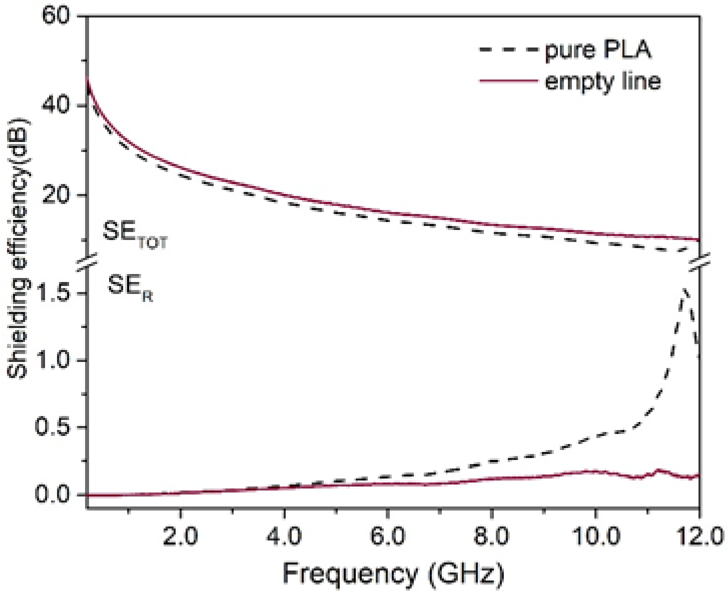

Figure S3. SE_{TOT} and SE_R spectra of pure PLA reference sample and an empty coax line.

To compliment the data in main manuscript, below we show the SE_{TOT} and SE_R full spectra for all GNP lateral sizes used in the study. Each sample was measured four times and the spectra shown below are the average of these measurements.

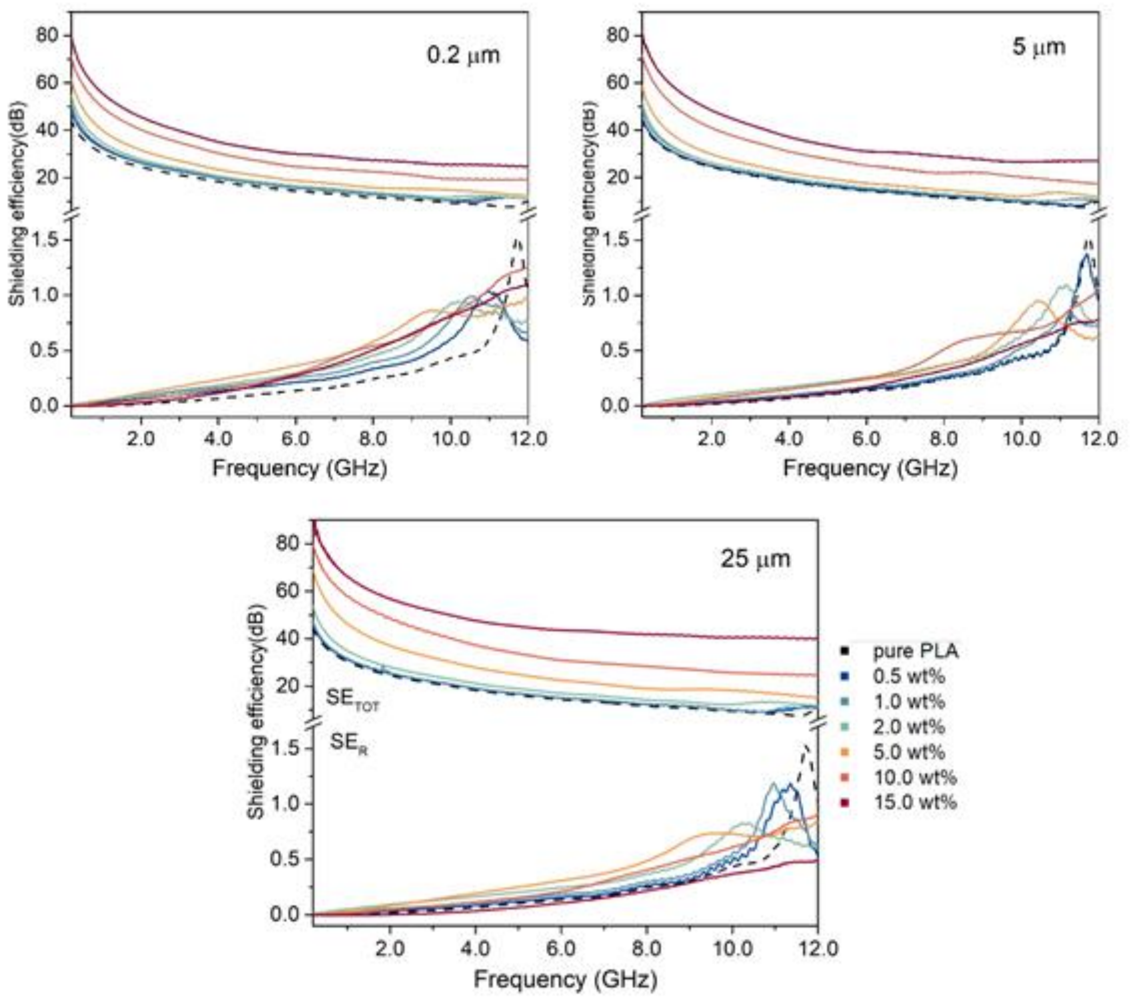

Figure S4. SE_{TOT} and SE_R of PLA/GNP composites with 0.2, 5 and 25 μm GNP lateral size, in function of frequency.

Electrical conductivity exponential fit

Below we show data for electrical conductivity σ presented in the main text with fitted curves and parameters of the exponential fit. The data points were fitted using following function $y = a\exp(bx)$. The fitted curves show good accordance with the experimental data, which is confirmed with the R-square test.

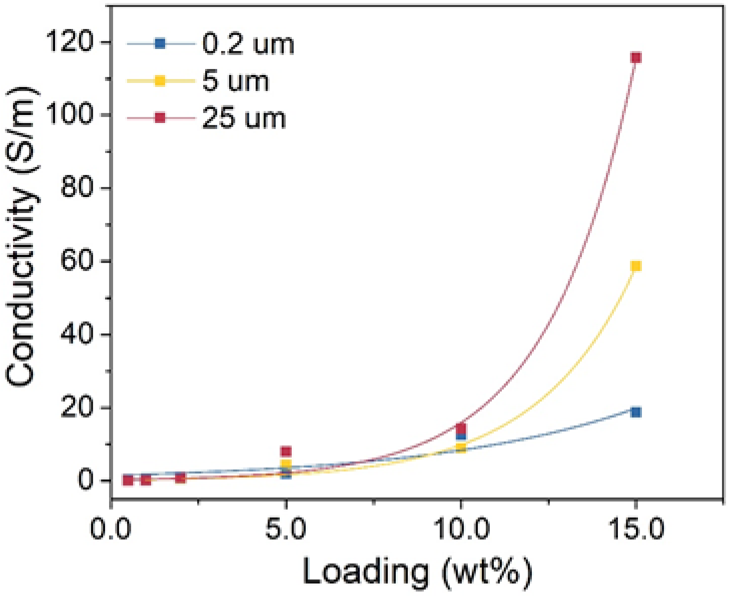

Model	Exp2PMod1		
Equation	y = a*exp(b*x)		
Plot	Conductivity	Conductivit	Conductivity
a	1.50389 ± 0.	0.279 ± 0.1	0.30203 ± 0.
b	0.17206 ± 0.	0.3564 ± 0.	0.3965 ± 0.0
Reduced C	6.67512	2.12926	9.06405
R-Square(	0.91606	0.99678	0.99652
Adj. R-Squ	0.89507	0.99598	0.99566

Figure S5. Experimental electrical conductivity data fitted with an exponential model, together with the fit parameters in the table.

Thermal conductivity exponential fit

Similar to the electrical conductivity, in Figure S6, we show data for thermal conductivity κ presented in the main text, together with fitted curves and parameters of the exponential fit. We have used the same fitting model as in σ. The quality of the fit is confirmed via the R-square test.

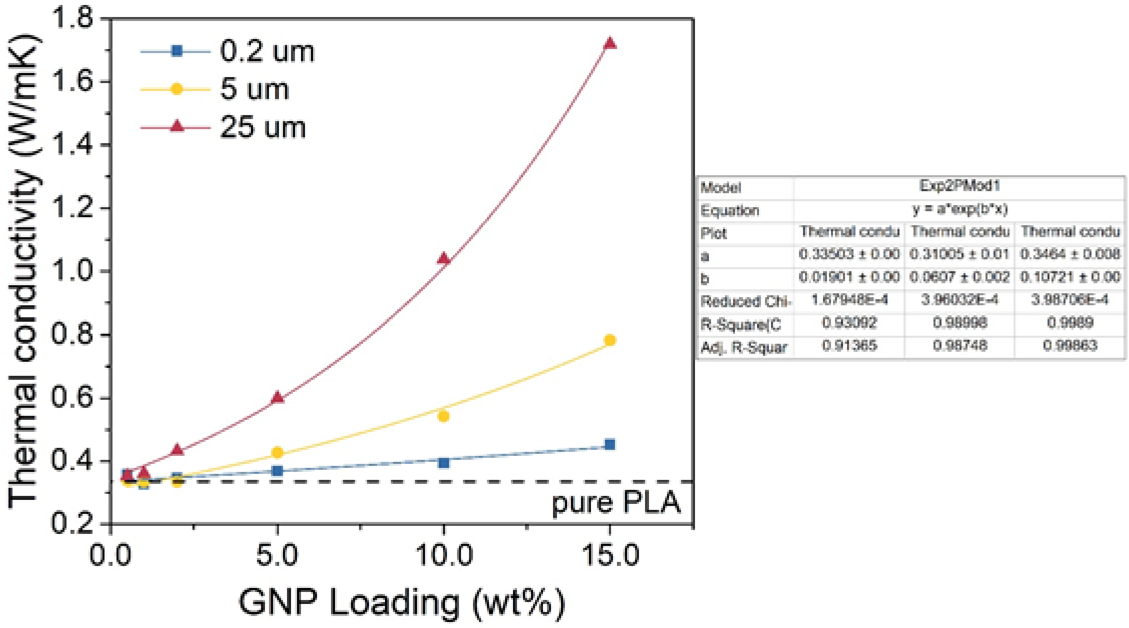

Model	Exp2PMod1		
Equation	y = a*exp(b*x)		
Plot	Thermal condu	Thermal condu	Thermal condu
a	0.33503 ± 0.00	0.31005 ± 0.01	0.3464 ± 0.008
b	0.01901 ± 0.00	0.0607 ± 0.002	0.10721 ± 0.00
Reduced Chi-	1.67948E-4	3.96032E-4	3.98706E-4
R-Square(C	0.93092	0.98998	0.9989
Adj. R-Squar	0.91365	0.98748	0.99863

Figure S6. Experimental thermal conductivity data fitted with an exponential model, together with the fit parameters in the table.
